# Development of PET Radioligands Targeting COX-2 for Colorectal Cancer Staging, a Review of *in vitro* and Preclinical Imaging Studies

**DOI:** 10.3389/fmed.2021.675209

**Published:** 2021-06-08

**Authors:** Caroline Dagallier, François Avry, Yann Touchefeu, Frédéric Buron, Sylvain Routier, Michel Chérel, Nicolas Arlicot

**Affiliations:** ^1^Unité de Radiopharmacie, CHRU de Tours, Tours, France; ^2^Inserm UMR1253, iBrain, Université de Tours, Tours, France; ^3^CRCINA, INSERM, CNRS, Nantes University, Nantes, France; ^4^Institut des Maladies de l'Appareil Digestif, University Hospital, Nantes, France; ^5^ICOA, Université d'Orléans, UMR CNRS 7311, Orléans, France; ^6^INSERM CIC 1415, CHRU de Tours, Tours, France

**Keywords:** COX-2, PET, colorectal cancer, radioligands, preclinical model

## Abstract

Colorectal cancer (CRC) is the second most common cause of cancer death, making early diagnosis a major public health challenge. The role of inflammation in tumorigenesis has been extensively explored, and among the identified markers of inflammation, cyclooxygenase-2 (COX-2) expression seems to be linked to lesions with a poor prognosis. Until now, COX-2 expression could only be accessed by invasive methods, mainly by biopsy. Imaging techniques such as functional Positron Emission Tomography (PET) could give access to *in vivo* COX-2 expression. This could make the staging of the disease more accurate and would be of particular interest in the exploration of the first metastatic stages. In this paper, we review recent progress in the development of COX-2 specific PET tracers by comparing the radioligands' characteristics and highlighting the obstacles that remain to be overcome in order to achieve the clinical development of such a radiotracer, and its evaluation in the management of CRC.

## Introduction

Colorectal cancer (CRC) is one of the most frequent types of cancer, ranking second in most developed countries, and has the second highest mortality rate (1, 2). This rate is especially high for late diagnosis and advanced stage disease. Thanks to an increase in CRC screening, the incidence rate has been decreasing for the last two decades (2). However, this high mortality rate draws early detection of CRC and prediction of recurrences and metastases as two major public health challenges, in order to initiate the appropriate treatment as early as possible.

Inflammation seems to play a key role in CRC physiopathology. Among the many known markers of inflammation, cyclooxygenase-2 (COX-2) was identified as having a crucial role from the first stages of tumorigenesis (3, 4). Reviews of over 30 epidemiologic studies showed that regular consumption of non-steroidal anti-inflammatory drugs (NSAID) was associated with a 30–50% reduced incidence of several cancer types, including CRC (5, 6). Interestingly, patients with familial adenomatous polyposis receiving COX-2 inhibitors as a preventive treatment developed fewer adenomas than patients receiving a placebo (7).

COX-2 is an enzyme that intervenes in the first steps of prostaglandin E2 synthesis from arachidonic acid and can be induced by various pro-inflammatory signals. COX-2 overexpression has been identified in different malignant neoplastic tissues, especially in up to 85% of adenocarcinomas (8–10) and is also associated with the potential for progression and recurrences of colorectal tumors. Molecular biology studies on CRC tissue samples concluded that COX-2 expression is significantly correlated with invasive (11) and metastatic phenotypes (12, 13). Moreover, high COX-2 expression in patients treated with radical surgery is a prognostic factor for recurrences, mainly because of undetected metastasis (13, 14). The COX-2 expression level is high in CRC cells. On the contrary, COX-2 has a low basal expression in healthy colon epithelial cells (15), making COX-2 a relevant biomarker for molecular imaging, especially for Positron Emission Tomography (PET).

The correlation of elevated COX-2 expression with the first stages of tumorigenesis and cancer progression suggests that COX-2 could be a target for the early imaging of pre-cancerous colorectal lesions. Since inflammation seems to be a hallmark of malignant CRC, PET imaging could allow the early detection of CRC dissemination and thus spot metastasis. In addition, in other cancerous pathologies (breast, prostate), COX-2 expression increases with the stage of the disease (16, 17). PET-scanning could therefore be a tool for CRC staging. Furthermore, positive therapeutic results in cohorts of patients with different types of solid tumors receiving COX-2 inhibitors in addition to their respective standard chemotherapy (18–20) suggest that COX-2 molecular imaging could provide stratification of the patients, enabling individualized treatment approaches. Notably, it could help to decide whether neoadjuvant chemotherapy is needed or determine whether patients would respond to COX-2 inhibitors therapy.

Up to now, numerous radioligands targeting COX-2 have been developed for SPECT or PET applications. Reviews by Laube et al., de Vries et al., and Pacelli et al., summarized their structures and synthesis methods and highlighted the challenges encountered in the development of COX-2 radiotracers (21–23). In the present review, we explore the recent progress of COX-2 molecular imaging in CRC, by comparing the radioligands' characteristics and highlighting the obstacles that remain to be overcome in order to achieve the clinical development of such a radiotracer.

The PubMed database was screened using pre-defined search dates (January 1995–January 2021). The search terms used were as follows: “colorectal cancer” and “COX-2” or “cyclooxygenase 2” and “PET.” It yielded 78 results. We screened preclinical results performed only on colorectal cancer cell lines. Title-, abstract- or full text-reading led to the exclusion of 70 papers because they did not focus on CRC or did not include *in vivo* PET radioligand evaluation or did not target COX-2. Eight original papers fulfilled these criteria and were thus included in the scope of the present review. Chemical structures of the PET radioligands are presented. The main conditions and results of these PET imaging preclinical studies assessing COX-2 radiotracers in CRC xenograft models are summarized in Table 1.

**Table 1:** Summary of characterization studies assessing COX-2 PET radiotracers in CRC xenograft models.

**Radioligand**	**Structure**	**Parent molecule**	***In vitro* models/cell lines**	**IC50 (μM)**	***In vitro* results**	***In vivo* model**	***In vivo* results**	**References**
^**18**^**F-3**Fluoromethyl-celecoxib	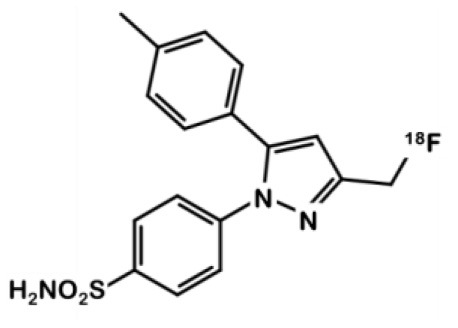	Celecoxib	*COX-2–*: HCT-116 COX-2+: RAW 264.7*	0.16	Affinity in the range of celecoxib's (IC_50_ = 0.03 μM). No blocking experiment available.	NU-Fox1nu mice bearing 1483 or HCT-116 cells	3-fold higher accumulation in the COX-2+ expressing tumor vs. COX-2- or muscle. Accumulation was inhibited by celecoxib pre-treatment	(24)
^**18**^**F-4**fluoromethyl-celecoxib derivative	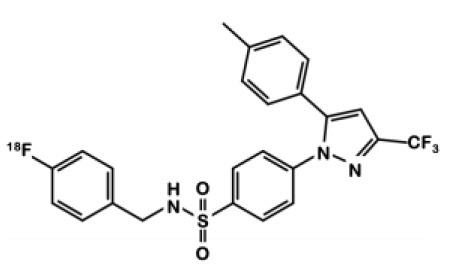	Celecoxib	COX-2+: HCA-7	0.36	Significant uptake after 60 min. No inhibition observed after pre-treatment with celecoxib	NIH-III mice bearing HCA-7 cells	Maximum T/M ratio of 1.4 10 min p.i. High intestinal uptake.	(25)
^**11**^**C-5**[11C]methoxy-1,2-diarylcyclopentane	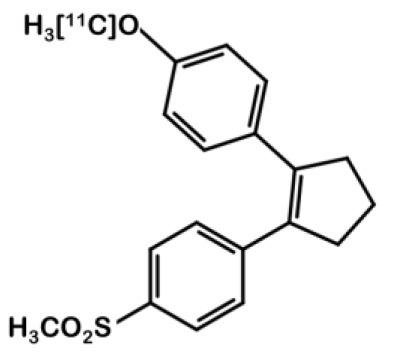	Coxib-like	*COX-2–*: RAW264.7 *COX-2+: HT-29*	0.005	High selectivity for COX-2 over COX-1 (x2000). In HT-29 cell cultures, Celecoxib pre-treatment reduced radioactivity uptake by 40% to 60%.	NMRI nu/nu mice bearing HT-29 cells	T/M ratio of 1.7 (60 min p.i.) Poor specificity (no effect of pre-treatment with a non-radioactive competitor). Fast metabolism (98 % eliminated 60 min p.i.). High intestinal and fat tissue uptake	(26)
^**18**^**F-6Pyricoxib**	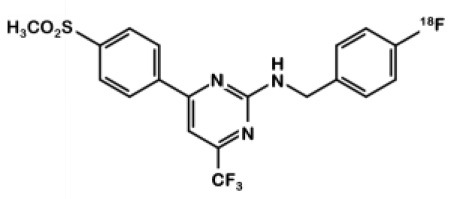	Celecoxib	*COX-2–*: HCT-116 *COX-2+: HCA-7*	0.007	Better *in vitro* affinity and specificity than its parent, celecoxib. Higher uptake in HCA-7 cells than HCT-116 cells. Maximum uptake reduction of 65% when pre-treated with non-radioactive coxib.	NIH-III nude mice bearing HCA-7 xenografts with or without celecoxib pre-treatment. Control group: NIH-III nude mice bearing HCT-116 cells.	50% decrease of radiotracer uptake in COX-2+ tumors after pre-treatment with celecoxib. T/M ratio of 2.25 (4 h p.i.) High intestinal uptake.	(27)
^**18**^**F-6-8**	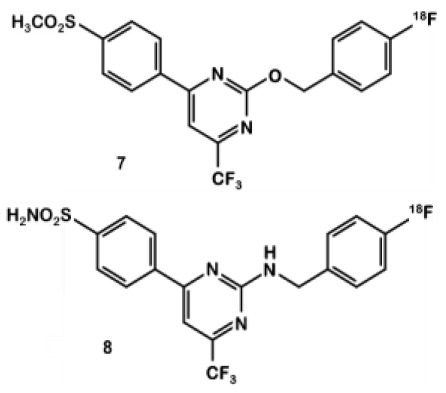	Celecoxib	*COX-2+*: HCA-7	**6**: 0.007**7**: 0.039**8**: 0.02	Similar *in vitro* properties (lipophilicity, affinity, specificity). Higher uptake for compound 6 > 7 > 8.	HCA-7 xenografts administered to NIH-III nude mice	PET acquisitions displated a substantially higher uptake of **[**^**18**^**F]6** than 7 and 8, with a T/M ratio of 1.19 (1 h p.i.). High intestinal uptake	(28)
^**18**^**F-9**Triacoxib	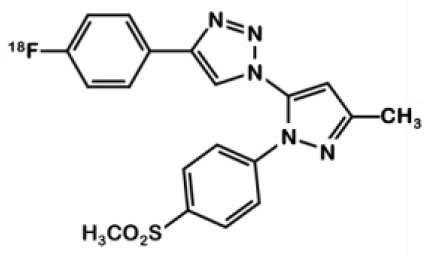	5-azido-pyrazole	*COX-2+*: HCA-7	0.09	COX-2/COX-1 selectivity ratio > 1,000. Pre-treatment with celecoxib induced a decrease of 47% in the uptake.	NIH-III mice bearing HCA-7 xenograft	Pre-treatment with celecoxib induced a decrease of 20% of the uptake. T/M ratio of 1.49 (1 h p.i.). Unspecific binding in lipid rich tissues.	(29)
^**18**^**F-10**	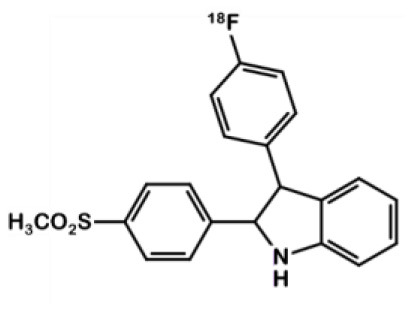	2,3-diaryl indole based on COX-2 inhibitors	*COX-2+*: HT-29	1.2	High uptake in COX-2+ cell lines. Pre-treatment with celecoxib induced a decrease of 80% in the uptake. Low specificity (COX-2/COX-1 ratio of 5.5);	NMRI nu/nu mice bearing HT-29 xenografts	No substantial accumulation of the radioligand in COX-2+ xenografts. High intestinal uptake.	(30)
^**125**^**I-12/**^**124**^**I-12indomethacin amide**	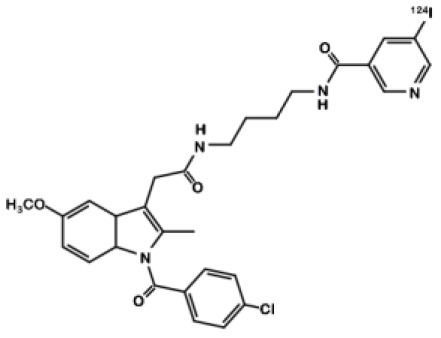	indomethacin	*COX-2* Celecoxib: HCT-116 COX-2+: HT-29, HEK, HUVEC	/	^**125**^**I-12** COX-2+/COX-2- uptake ratio of 7.6 Pre-treatment with celecoxib induced a decrease of 70% in the uptake.	HT-29 and HCT-116 xenografted SCID mice	^**124**^**I-12** Normalized uptake (kBq/g tissue) was approximately 5-fold higher in HT-29 tumors than in HCT-116 tumors. T/M ratio was more than 50-fold higher in HT-29 xenografts compared to HCT-116 xenografts. μPET imaging highlighted a predominantly intestinal uptake (no quantitative results available).	(31)

The table summarizes for each probe: chemical structure, parent molecule, IC_50_, cell lines used for in vitro studies and conclusion of these studies, models used for preclinical studies and conclusions, reference article. Cell lines with high COX-2 expression: HT-29, Human colorectal adenocarcinoma cells; HCA-7, Human colon adenocarcinoma cells; HUVEC, human umbilical vein endothelial cell; HEK, human embryonic kidney. Cell lines with low COX-2 expression: HCT-116, human colorectal carcinoma cells; ^*^RAW264.7, murine macrophage cell line. Low basal COX-2 expression, but that can be induced, for instance with γ-interferon and lipopolysaccharide.

## Coxib Family Derivatives

**Figure 1:**
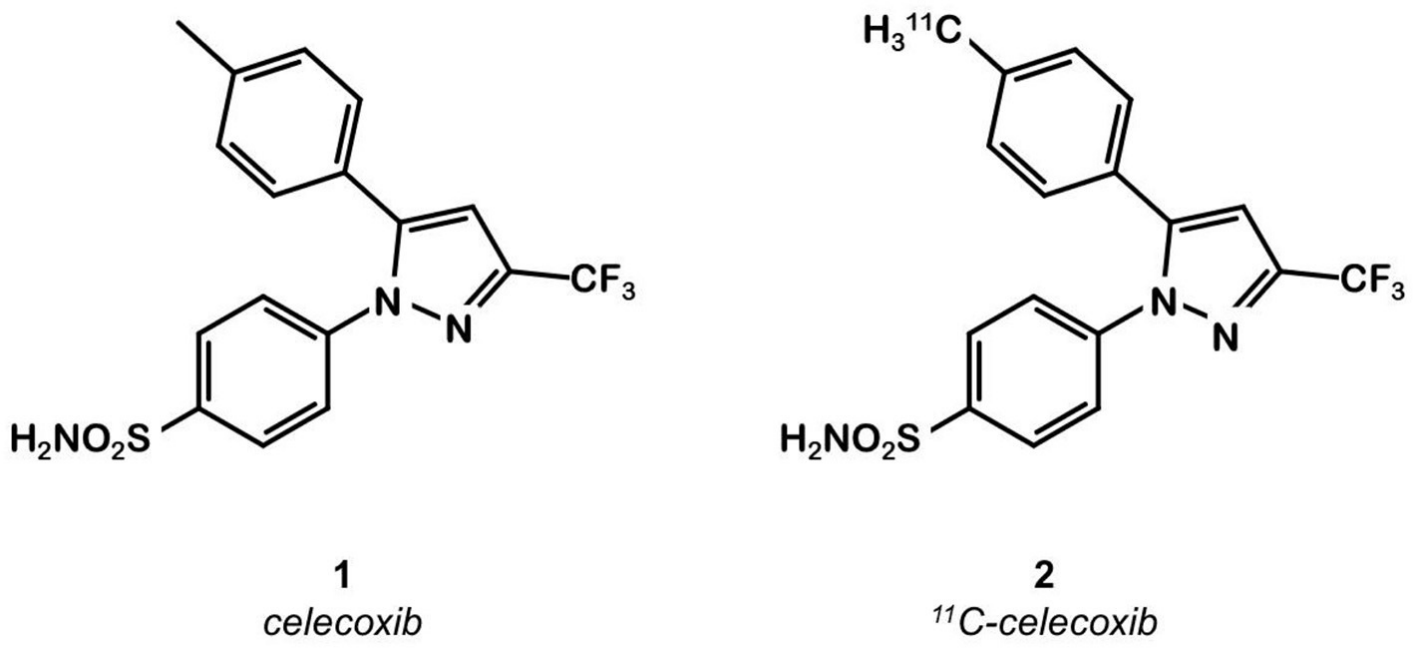
Celecoxib and ^11^C-celecoxib structures.

**Figure 2:**
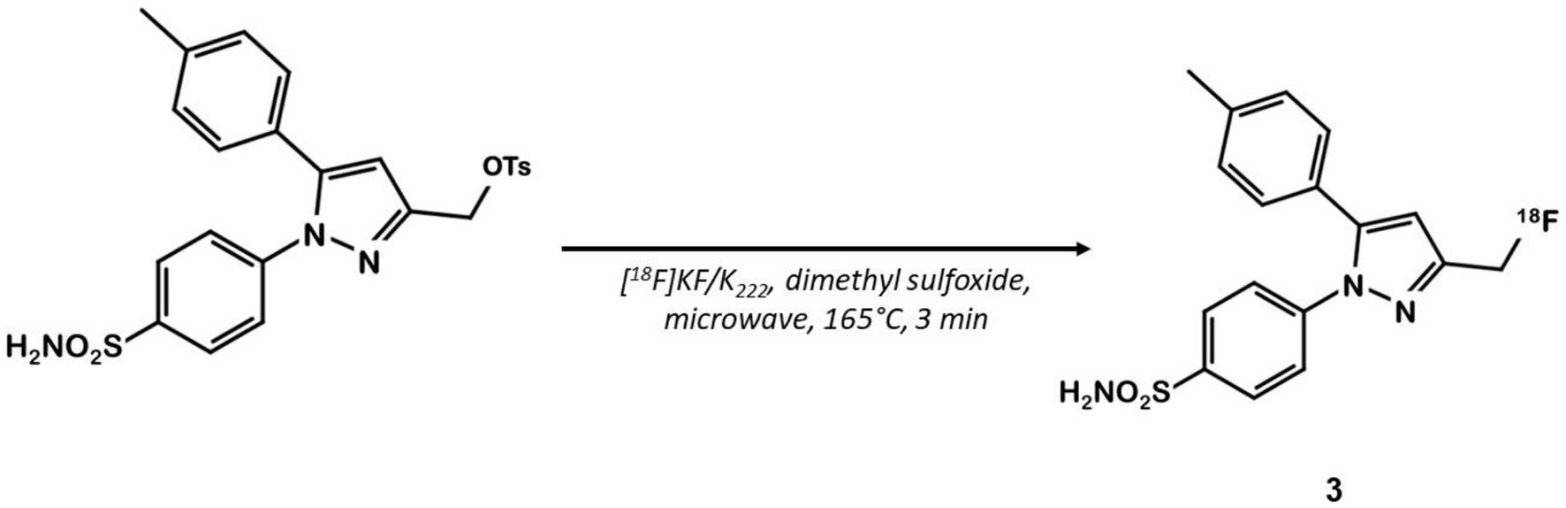
Radiosynthesis of pyrazole derivative ^18^F-3.

Celecoxib **1** (Figure 1) is a well-known COX-2 specific inhibitor used as an anti-inflammatory drug. Its specificity and selectivity for COX-2 over COX-1 makes it a natural candidate for PET applications. In 2005, Prabhakaran et al. synthesized ^11^C-Celecoxib **2** (Figure 1) in 8 efficient steps including a palladium catalyzed radiomethylation (32), but this radioligand has not yet been tested in a CRC preclinical model. A biodistribution study in baboons showed unsatisfactory pharmacokinetic properties. ^11^C-**2** underwent a fast metabolism (80% after 30 min in plasma samples) and was quickly cleared out of the organism by urinary excretion (33). Therefore, several series of celecoxib derivatives were proposed to work around the limitations of celecoxib. In 2011, Uddin et al. developed a series of celecoxib [^18^F]-fluorine derivatives (24). Synthesis in 7 steps involving an aliphatic nucleophilic substitution led to the best candidate. Diethylaminosulfurtrifluoride (DAST) mediated fluoration of its alcohol precursor gave the fluoromethyl derivative ^18^F-**3** (Figure 2). In inhibition assays using purified COX-2, ^18^F-**3** exhibited an IC_50_ of 160 vs. 30 nM for **1**. *In vivo* evaluation was conducted in a Nu-fox1nu mice model bearing human head and neck squamous cell carcinoma cells HNSCC 1483 (COX-2+) or human colorectal carcinoma cells HCT-116 (COX-2–). A biodistribution study gave promising results, with an uptake ratio in COX-2 positive/COX-2 negative tumors of 3, similar to the COX-2+ tumor to muscle ratio (T/M). Blocking experiments with cold celecoxib produced a clear decrease of the radiotracer uptake in the COX-2+ tumor (T/M = 1). However, only the COX-2 negative tumors were CRC cells (HCT-116); the COX-2 positive model was a xenograft of human head and neck squamous cell carcinoma.

**Figure 3:**
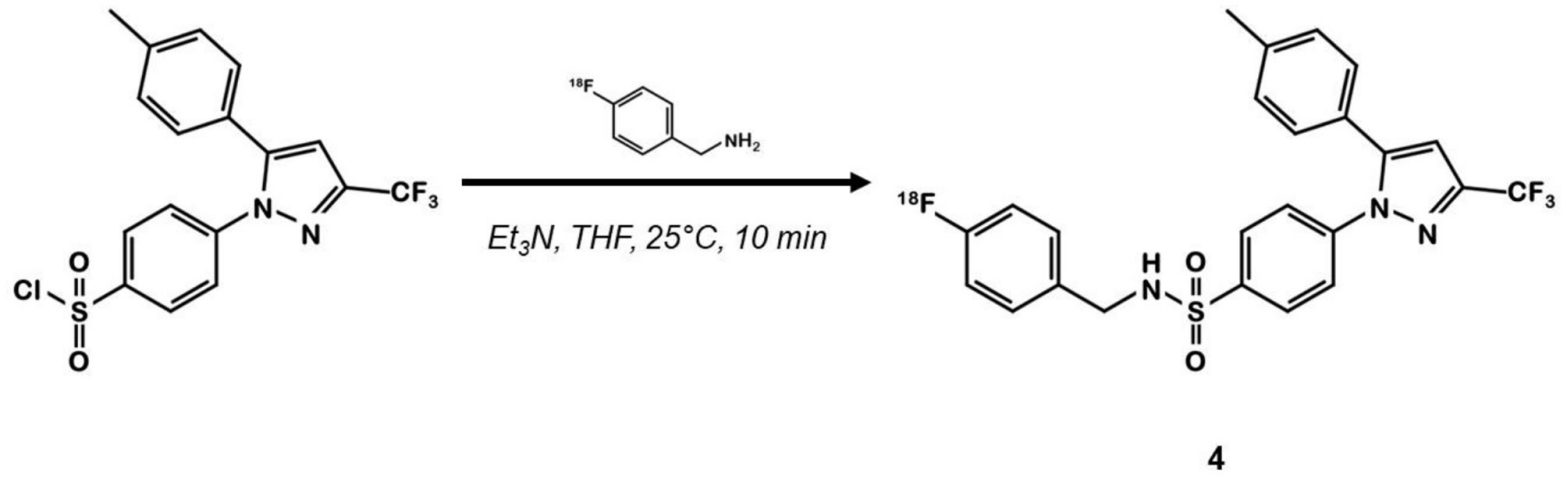
Radiosynthesis of pyrazole derivative ^18^F-4.

In 2015, Kaur et al. synthesized a radiolabeled derivative ^18^F-**4** from a key sulfonylchorine and its amination by the prosthetic 4-[^18^F]fluorobenzylamine scaffold (25) (Figure 3). Derivative **4** exhibited interesting *in vitro* properties on human recombinant COX-2 (log P = 3.18; IC_50_ = 360 vs. 40 nM for **1**) but suffered from a lack of specificity. Indeed, cellular uptake experiments in human CRC cells expressing COX-2 (HCA-7) showed an accumulation of the radioligand that could not be inhibited by a celecoxib or rofecoxib pre-treatment. *In vivo*, NIH-III mice xenografted with HCA-7 cells exhibited a maximum tumor to muscle ratio 10 min post injection (p.i.) of 1.4, slowly decreasing over time. Dynamic acquisitions showed a rapid elimination of the radioactivity through the intestinal tract. *In vitro* results were not convincing, and specificity was not investigated *in vivo*.

**Figure 4:**
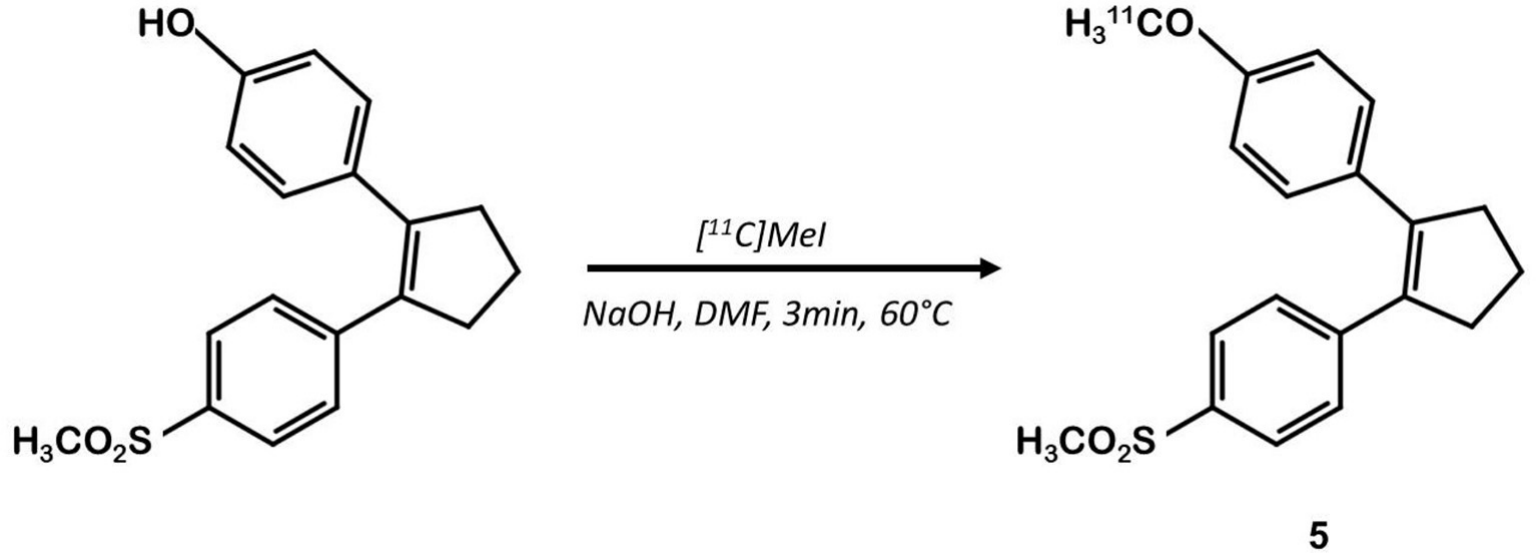
Radiosynthesis of [^11^C]diarylcyclopentene 5.

Other teams focused on different coxib-like derivatives. Derivative **5** was first studied by Li et al. and exhibited a very promising COX-2 affinity, with an IC_50_ in the range of the nanomolar concentration (IC_50_ = 5 nM) and a high *in vitro* selectivity for COX-2 over COX-1 (COX-1/COX-2 = 2,000) (34). Wuest et al. synthetized the [^11^C]methoxy-1,2-diarylcyclopentene ^11^C-**5** in 4 steps from 2-dibromocyclopentene using a double Suzuki cross coupling and as final step a Williamson type O-methylation with [^11^C]CH_3_I (26) (Figure 4). The authors confirmed the specificity of compound **5** for COX-2 with cellular uptake studies (especially in the COX-2+ human colorectal adenocarcinoma cell line HT-29 vs. COX-2- RAW264.7). Its lipophilicity of 4.2 enabled it to cross the cell membranes but might be responsible for a lack of specificity *in vivo*. In fact, *in vivo* assays on a preclinical rodent xenograft model of human CRC cells (HT-29 cells, COX-2+) exhibited an accumulation of the tracer in the tumor, that could not be inhibited in blocking experiments with an excess of non-radioactive competitor. Moreover, this radiotracer was quickly metabolized, with only 17% remaining intact in plasma samples 10 min p.i.

**Figure 5:**
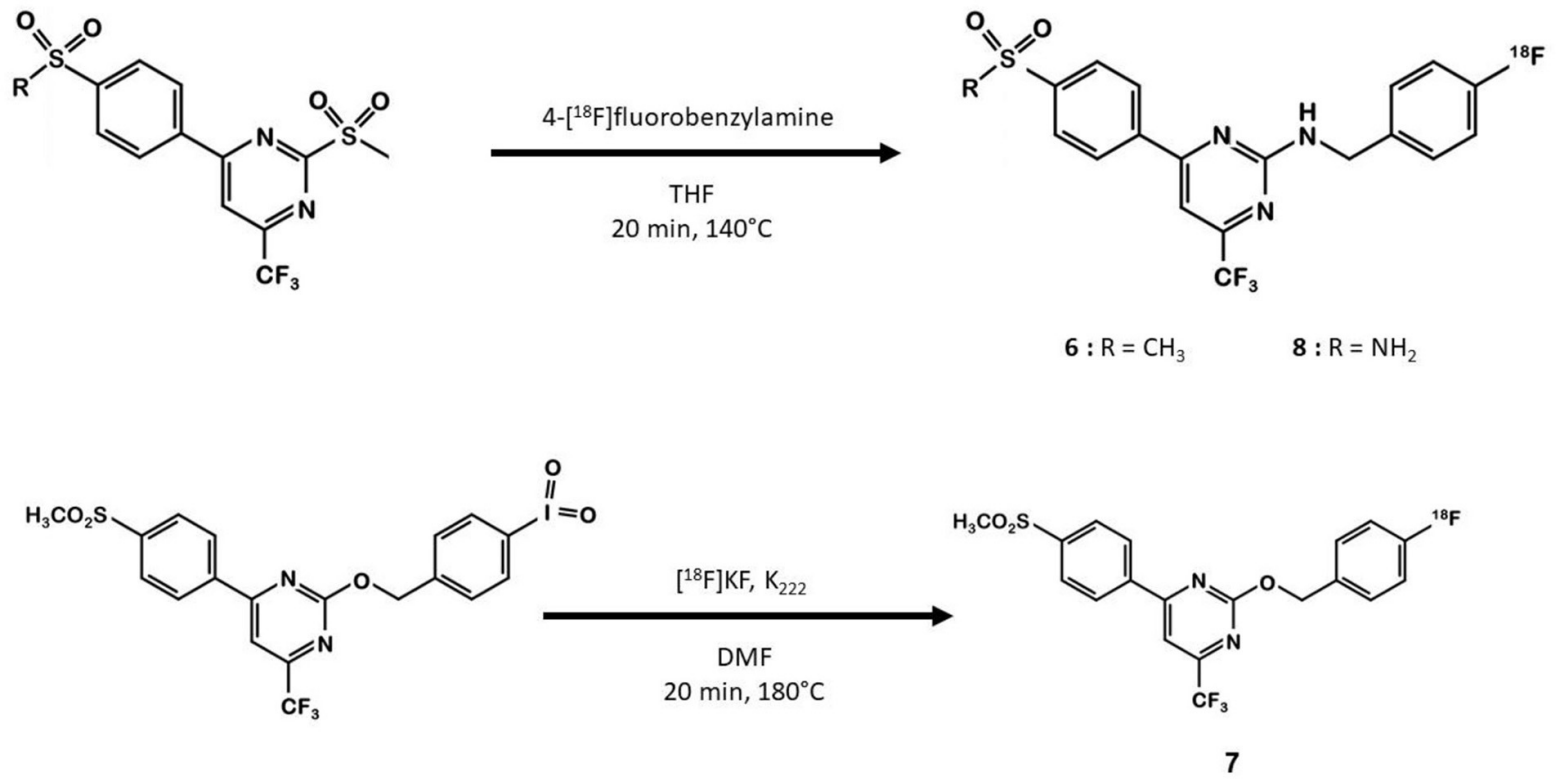
Radiosynthesis of ^18^F-6-8 probes.

In 2013, Tietz et al. developed ^18^F-Pyricoxib **6**, designed by replacing the celecoxib pyrazole ring by a pyrazine isoster (35). In 2016, Tietz et al. proved that derivative **6** showed better *in vitro* affinity and specificity than its parent Celecoxib **1** (IC_50_ = 7 vs. 40 nM for **1**) (27). *In vitro* blocking experiments in HCA-7 and HCT-116 cells were promising. Radiotracer uptake in COX-2 positive cells (HCA-7) was significantly higher than in COX-2 negative cells (HCT-116), and pre-treatment with diverse non-radiolabeled COX-2 inhibitors led to a maximum uptake reduction of 65%. *In vivo* experiments were then conducted on NIH-III mice xenografted with either HCA-7 or HCT-116 cells, showing a satisfactory uptake in HCA-7 tumors (T/M ratio of 2.25 after 4 h p.i.). Pre-treatment with 2 mg of Celecoxib **1** (intraperitoneal administration, i.p.) led to a marked decrease in radiotracer uptake in the tumors. Biodistribution results showed a %ID/g of 2.12% in HCA-7 tumors, scaled down to 1.04% after a celecoxib pretreatment. However, radiotracer uptake was similar in HCA-7 and HCT-116 tumors. Expression of COX-2 in HCT-116 tumors was confirmed by immunohistochemistry, highlighting a cell line drift and questioning the relevance of the HCT-116 cell line as a COX-2 negative model. They concluded that ^18^F-pyricoxib **6** was a very promising candidate for a “first in human” study (27). In 2018, Tietz et al. deepened their understanding of this chemical entity by performing pharmacomodulation experiments (28). For the O-analog ^18^F-**7** the radiolabeling was carried out on a preoxidized iodyl precursor. The authors started from 4-chloro-2-(methylsulphonyl)-6-(trifluoromethyl)pyrimidine after 5 steps including radiolabeling with 4-[^18^F]fluorobenzylamine. ^18^F-**8** radiosynthesis was similar to that of ^18^F-pyricoxib **6**, using sulfonyl precursors and a 4-[^18^F]fluorobenzylamine (FBA) as building block (Figure 5). The three tested probes ^18^F-**6-8** exhibited similar *in vitro* properties (lipophilicity, affinity, specificity for COX-2). However, even slight modifications of the structure led to radically different pharmacokinetic, biodistribution and uptake profiles (Table 1). One of the radiotracers was slightly metabolized and excreted *via* the urinary tract but did not accumulate in the tumors, while the other was rapidly eliminated but had a better T/M ratio.

**Figure 6:**
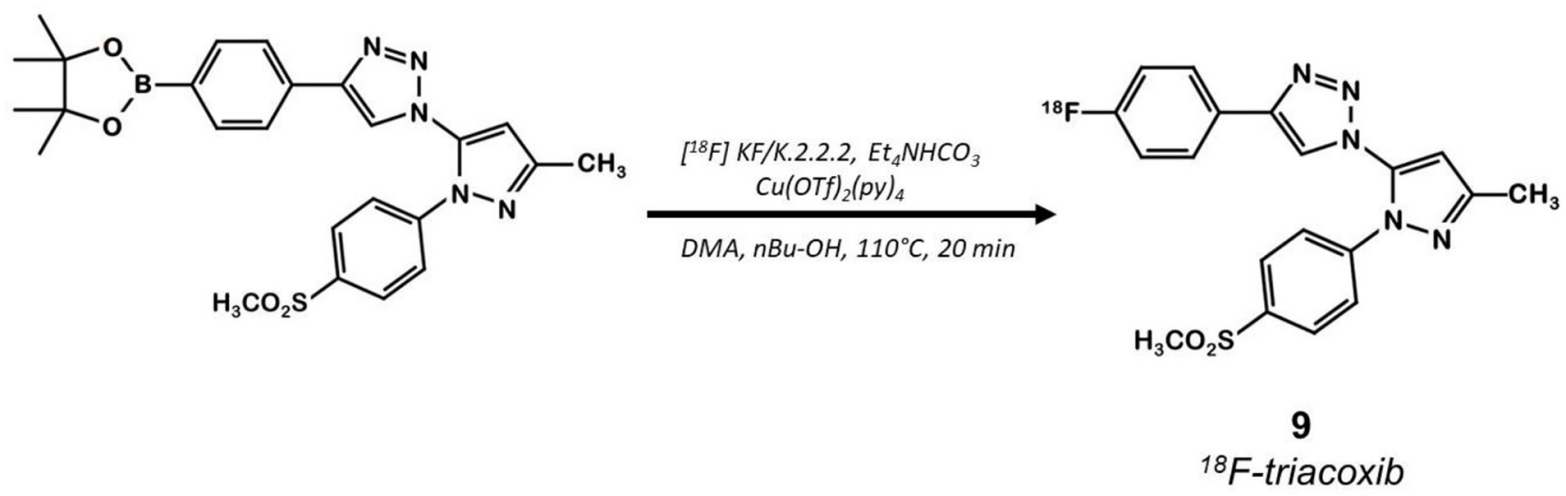
Radiosynthesis of ^18^F-Triacoxib 9.

The same team developed a pyricoxib derivative to reduce this unspecific binding previously observed with ^18^F-pyricoxib **6** (29). They designed ^18^F-triacoxib **9** which was obtained from the Celecoxib **1** structure by replacing the CF_3_ by a CH_3_ group and the acidic sulfonylamide by a simple methylsulfonyl. Nevertheless, the best innovation in this series consisted in the insertion of a triazole between the pyrazole and the phenyl ring, which required building an azidopyrazole as key intermediate. Noteworthy, ^18^F-fluorination was achieved using a pinacol boronic ester (in the presence of a copper catalyst [Cu(OTf)_2_(py)_4_]) as leaving group (Figure 6). Compound **9** displayed a satisfactory *in vitro* affinity and selectivity for COX-2 (IC_50_ = 90 vs. 70 nM for **1**; IC_50_ COX-1 > 100 μM for both compounds, HCA-7 cells). *In vitro* binding experiments with increasing doses of non-radiolabeled compounds (celecoxib or triacoxib) resulted in an inhibition of the radiotracer uptake by 47 to 63%. *In vivo* experiments on BALB/c mice bearing HCA-7 xenografts suggested an improved stability of ^18^F-triacoxib **9** compared to ^18^F-pyricoxib **6** (respectively, 90 vs. 75% intact radiotracer in plasma samples 60 min p.i.). PET acquisitions confirmed a higher uptake in the tumors than in the muscle, within a similar range as ^18^F-pyricoxib **6** (tumor-to-muscle ratio of 1.49 vs. 1.47, respectively, 60 min p.i.). In Celecoxib **1** pretreated mice, a partial inhibition of ^18^F-triacoxib **9** uptake was observed (20%). Unspecific binding was also observed in lipid rich tissues. ^18^F-triacoxib **9** did not seem to be a better candidate than ^18^F-pyricoxib **6** as a diagnostic tool for CRC.

## 2,3-Diaryl Indoles

**Figure 7:**
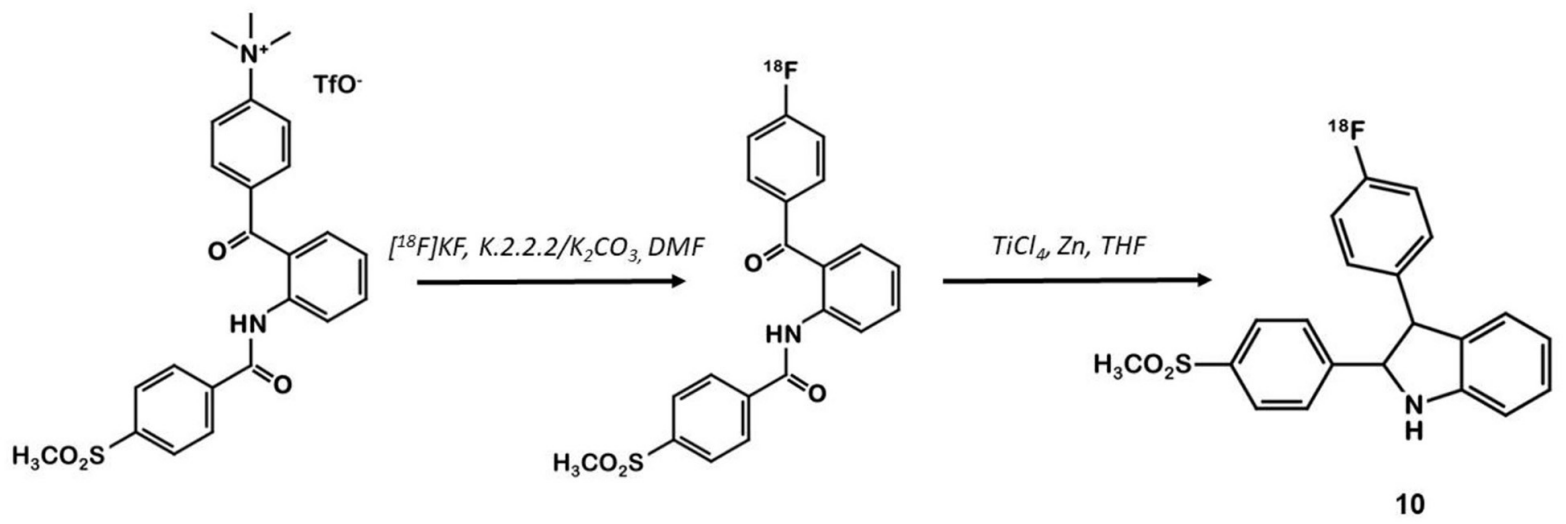
Radiosynthesis of ^18^F-10 via McMurry cyclisation.

In 2003, Hu et al. synthesized a large series of original 2,3-diaryl indoles (36). Structure-Activity Relationships (SAR) were established from about thirty derivatives obtained from anthranilic acids in 5 or 6 steps including indole formation by McMurry cyclization (Figure 7). Derivative **10** exhibited high affinity and selectivity for COX-2 (IC_50_ = 20 vs. 520 nM for **1**) over COX-1 (IC_50_ >10 μM for both compounds), and better anti-inflammatory properties than celecoxib in a rat carrageenan-induced foot pad edema assay. In 2012, Kniess et al. radiolabeled this promising compound using the nucleophilic substitution of a trimethylammonium salt by ^18^F-fluorine (30). They investigated the determination of PGE2 levels in cell culture supernatants, which is therefore a direct measure of the COX activity. The cellular inhibition assay results demonstrated **10** to be a potent cyclooxygenase inhibitor with only low COX-1/COX-2 selectivity. The authors evaluated the selectivity of **10** using an enzymatic competitive inhibition assay with celecoxib as reference. IC_50_ = 6.6 μM for COX-1 and 1.2 μM for COX-2 were determined (IC_50_ COX-1 = 115 and 0.06 μM for **1**). Pre-incubation of HT-29 cells with cold compound reduced the cellular uptake by almost 80% compared to incubation with the radioactive compound alone. *In vivo* PET imaging of HT-29 xenografted mice showed no accumulation of the tracer in the tumor. Despite a low lipophilicity (log D = 1.2), the tracer was rapidly eliminated through the intestinal tract (half-life: 8 min).

## Indomethacin Derivatives

**Figure 8:**
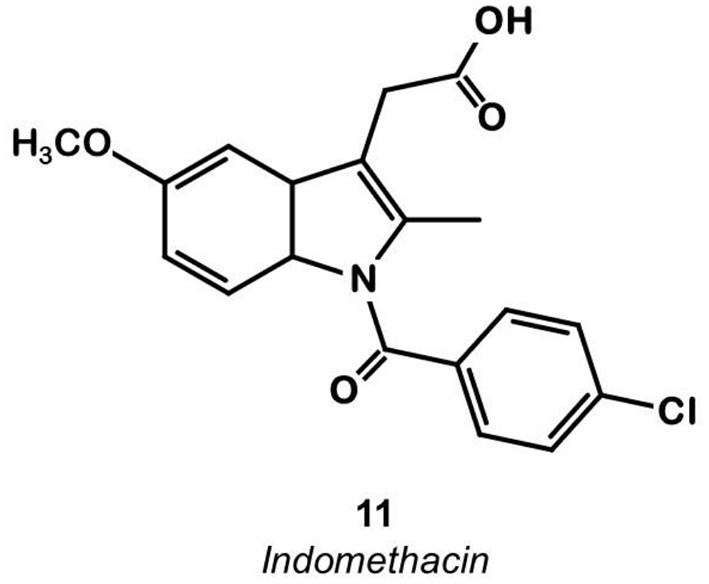
Indomethacin.

**Figure 9:**
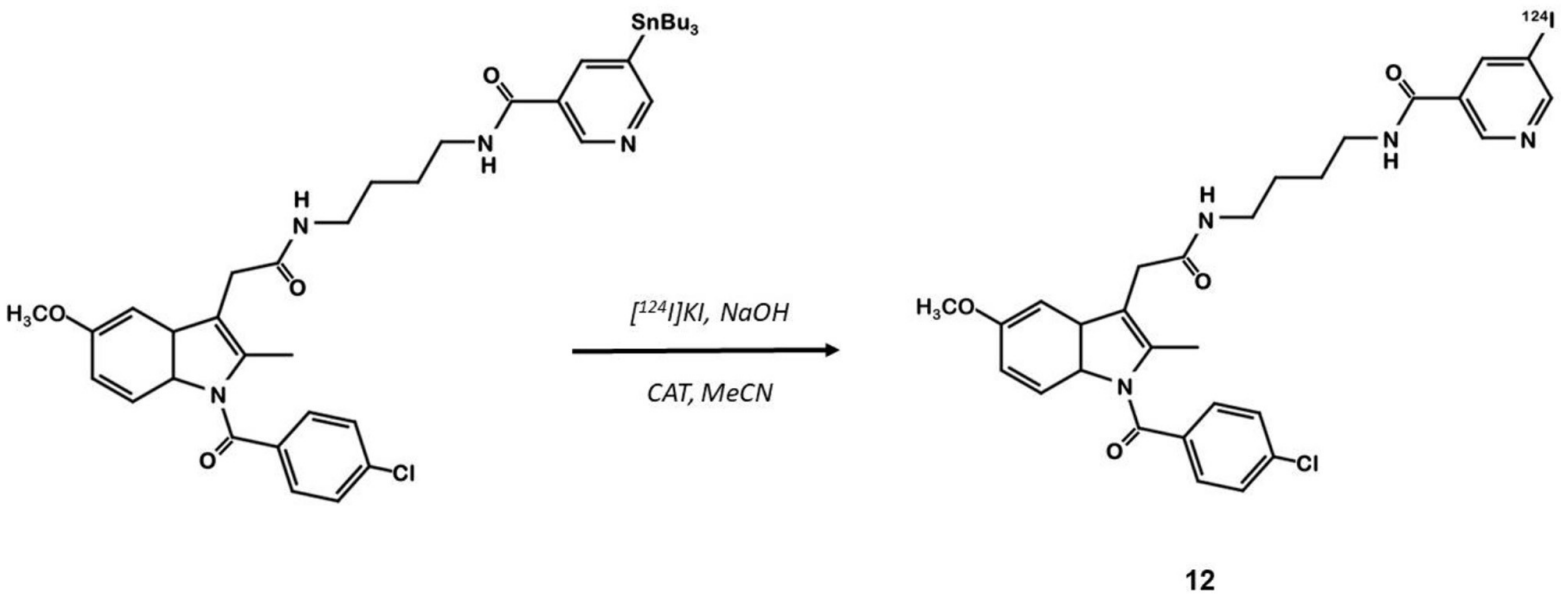
Radiosynthesis of indomethacin derivative ^124^I-12.

Indomethacin **11** (Figure 8) is a COX ligand that binds to both COX-1 and COX-2. Kalgutar et al. modified its chemical structure by adding an amide group (37). After SAR analysis and the building of nearly twenty analogs, they clearly improved the selectivity of the derivatives for COX-2, which was inhibited in the nanomolar range. Following this work, Morgenroth et al. synthetized radio iodinated analogs from stannyl precursors (31). Derivative ^125^I-**12** (Figure 9) was found to be the most suitable tracer. *In vitro* studies on CRC cell cultures HT-29 and HCT-116 showed a significantly higher concentration of **12** in COX-2 positive (HT-29) cells (COX-2+/COX-2– ratio of 2.5 and 7.6 for the two tested radiotracers). Blocking experiments were performed on human embryonic kidney (HEK) and human umbilical vein endothelial cell (HUVEC) cell lines, showing a partial decrease of the uptake in cells pre-treated with celecoxib **1**. These affinities and selectivities were confirmed using both *in vivo* microPET and bio-distribution studies. The compound with the highest uptake was radiolabeled with ^124^I and used for PET imaging of HT-29 and HCT-116 xenografted mice. Due to the lipophilicity of this compound (log D = 4.41), the radioactivity was mainly localized in the liver and the gastrointestinal tract. No tracer uptake was observed in HCT-116 tumors whereas HT-29 tumors displayed a significant uptake. Measurement of the remaining activity in each organ with a gamma counter confirmed a COX-2+/COX-2– ratio of 5. The T/M ratio was over 50 times higher in HT-29 tumors than in HCT-116 tumors.

In 2011, Uddin et al. also synthesized a series of fluorine-containing indomethacin derivatives. These probes were satisfactory COX-2 inhibitors with an IC_50_ in the range of the nanomolar both in inhibition assays using purified COX-2 and intact cells. However, they could not be successfully radiolabeled due to a lack of stability in the severe reaction conditions required (24).

## Discussion

This review focuses on COX-2 radiotracers tested in CRC models. Other COX-2 radiotracers have also been developed for other oncologic indications as well as applications in the field of neuroinflammation (22).

**Figure 10:**
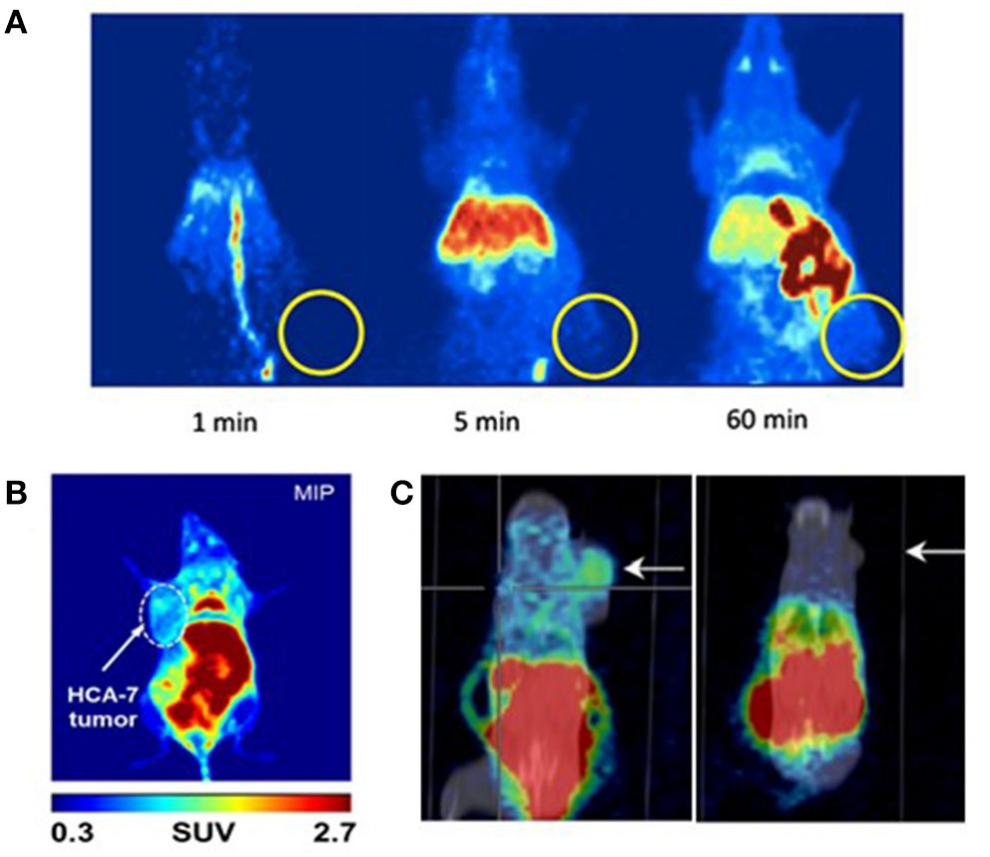
microPET images of mice xenografted with human colorectal cancer cell lines after injection of a COX-2 specific radiotracer. **(A)** Kniess et al., 2012 (30): Maximum intensity projection (MIP) images at 1, 5, and 60 min p.i. after a single IV injection of 18F-3 into HT-29 tumor-bearing (right flank) NMRI nu/nu mice. Authors concluded that ^18^F-**3** was not promising. Reprinted from Bioorg Med Chem. 2012 Jun 1;20(11):3410–21, Radiosynthesis of a 18F-labeled 2,3-diarylsubstituted indole via McMurry coupling for functional characterization of cyclooxygenase-2 (COX-2) *in vitro* and *in vivo*. Kniess T, Laube M, Bergmann R, Sehn F, Graf F, Steinbach J, et al. Copyright (2012), with permission from Elsevier, license number 5067540791584. **(B)** Litchfield et al. (29): Maximum intensity projection (MIP) images at 60 min p.i. of ^18^F-**9** into HCA-7 tumor-bearing (left flank) BALB/c mice. Authors concluded that ^18^F-**9** was promising. **(C)** Morgenroth et al. (31): μPET/CT molecular imaging of COX-2 with ^124^I-**12** in HT29 (left panel) and HCT-116 (right panel) xenografted SCID mice at 4 h p.i. Arrows indicate tumor. Authors concluded that ^124^I-**12** was promising.

These eight studies on CRC xenografts display heterogeneous results. Kaur et al. (25), Wuest et al. (26), and Kniess et al. (30) concluded that their radiotracer was not appropriate for further clinical investigation, whereas Uddin et al. (24), Tietz et al. (27, 28), Litchfield et al. (29), and Morgenroth et al. (31) judged their radiotracer to be promising for clinical applications (satisfactory T/M ratio). However, for all the tested compounds, most of the radioactivity uptake was located in the digestive tract. The T/M ratios were deemed satisfactory, ranging from 1.4 to 5.0, but the tumor-to-intestine ratio was not always mentioned in these studies, and, when available, was well above the T/M ratio. The tumors were detectable as xenografts in mice flanks, but these T/M ratios would probably not be sufficient to detect *in situ* CRC tumors (Figure 10). Thus, the challenge to develop the ideal COX-2 radiotracer for CRC staging remains to date. Two areas of work can be explored to achieve this radiotracer development for CRC imaging.

### Radiotracer-Linked Properties

All the tested compounds display a high lipophilicity (log P or log D ϵ [1.7–4.4]), required to reach their intracellular target COX-2, but also responsible for hepatobiliary excretion that could mask CRC tumors. This assumption questions the relevance of COX-2 as a CRC imaging target and could be tested using *in situ* CRC preclinical models. ^11^C-celecoxib was deemed unfit for clinical applications but benefited from a renal excretion that would be a serious advantage in CRC imaging. To increase the affinity for COX-2, pharmacomodulations on this parent compound led to more lipophilic molecules. There is still a need to balance affinity and lipophilicity with ideal pharmacokinetic properties. The use of innovative vectors could prove to be the solution to selectively deliver radiolabeled coxib derivatives into cancerous cells. In particular, the use of the RGD sequence (a tripeptide composed of arginine, glycine and aspartic acid) (38), dendrimers (39) or dehydropeptides (40) proved their potential to carry COX inhibitors into inflammatory or cancerous cells. Other pharmacokinetic properties should be taken into account, especially the metabolization rate and the radiotracer half-life. To be used routinely, the ideal radiotracer should be more than 50% intact 1 h p.i. and the half-life of the vector should not be shorter than 30 min. Other well-known PET tracers, such as ^18^F-FDG or ^18^F-FDOPA comply with these requirements (respective renal excretion of 20% 2 h p.i.^1^ and 50% 45 min p.i.^2^).

The second parameter to consider is the radioisotope. Due to its 110 min half-life, the use of ^18^F is widespread in PET imaging, and most of the COX-2 binders were labeled with ^18^F. Morgenroth et al. obtained the maximum T/M with a radio-iodinate derivative (31). However, due to both the higher energy of emitted beta particles and the longer half-life of ^124^I (4.2 days), radio-iodinate derivatives exhibit a poorer dosimetry profile compared to ^18^F-compounds. On the contrary, the shorter half-life of ^11^C makes it less accessible for routine use (on-site cyclotron required) but would endow it with a better dosimetry profile.

Finally, pharmacomodulations were performed to increase the specificity for COX-2 compared to COX-1, but other targets are known for coxibs. For instance, celecoxib or valdecoxib bind significantly (IC_50_ in the order of nM) to carbonic anhydrase isoenzymes (CA) (41). In addition, celecoxib is able to bind to PDK1 (42, 43), a cell survival regulation enzyme *via* the Akt/PKB pathway, with an IC_50_ in the range of μM (44). Likewise, coxibs bind to the transmembrane protein SERCA (45–47), a pump that can induce cellular apoptosis by increasing the intracellular calcium concentration. These different pro-apoptotic and anti-oncogenic targets have been confirmed by an increasing number of reports indicating that celecoxib does not require the presence of COX-2 to exert its anti-tumoral activity (44, 48, 49). Even more striking, it has been shown that structural analogs close to celecoxib, devoid of any COX-2 inhibitory activity, were able to mimic the anti-tumor properties of celecoxib studied so far, not only *in vitro* but also in various *in vivo* xenograft models (43, 50, 51). These data question the relevance of radiolabeled coxibs as specific COX-2 binders.

### *In vivo* Xenograft Models

Xenografts are known to lead to derangement of the normal tumor architecture and nearby healthy tissues and vasculature, and cause altered drug-sensitivity (52, 53). To address these possible biases, the use of orthotopic xenograft or genetically engineered mice expressing human colorectal cancer genes would mimic *in situ* CRC more accurately (54), easing the evaluation of the impact of unspecific intestinal uptake. Imaging the xenografts with ^18^F-FDG prior to the radiolabeled COX-2 inhibitor would also inform about the accessibility of tumors for PET radioligands, notably regarding their perfusion.

## Conclusion

Ultimately, most of these radioligands exhibit promising affinity and specificity *in vitro* but fail to prove their efficiency *in vivo* on xenograft models. Chemical screening and pharmacomodulations have yet to work out the ideal COX-2 radiotracer. The use of innovative vectors should be considered to selectively deliver radiotracers in the tumors. Given the hepatobiliary excretion of most of the known coxib derivatives, *in situ* CRC models should be considered for future explorations. *In vivo* preclinical studies on *in situ* CRC models would be decisive to conclude whether COX-2 is a relevant target in CRC imaging. More conclusive *in vivo* results are required before conducting a “first-in-human” study.

## Author Contributions

CD wrote the present paper and contributed to the design of the review. FA contributed to the redaction of the manuscript and to the prior bibliographic study. YT contributed to the clinical rational of the work. FB and SR wrote chemical sections of the manuscript. MC contributed to manuscript revision and improvement. NA designed the objectives of the review and supervised its preparation and redaction. All authors have substantially contributed to this work. The manuscript has been re-read and approved by all authors, and they have all contributed to its scientific improvement.

## Conflict of Interest

The authors declare that the research was conducted in the absence of any commercial or financial relationships that could be construed as a potential conflict of interest.

## Footnotes

^1^Résumé des Caractéristiques du Produit FDG. Available online at: http://agence-prd.ansm.sante.fr/php/ecodex/rcp/R0187540.htm (accessed June 9, 2020).

^2^Résumé des Caractéristiques du Produit FDOPA. Available online at: http://agence-prd.ansm.sante.fr/php/ecodex/rcp/R0157439.htm (accessed October 6, 2020).
